# Changes in Quantity and Sources of Dietary Fiber from Adopting Healthy Low-Fat vs. Healthy Low-Carb Weight Loss Diets: Secondary Analysis of DIETFITS Weight Loss Diet Study

**DOI:** 10.3390/nu13103625

**Published:** 2021-10-16

**Authors:** Lisa C. Offringa, Jennifer C. Hartle, Joseph Rigdon, Christopher D. Gardner

**Affiliations:** 1 Stanford Prevention Research Center, Stanford University School of Medicine, Stanford, CA 94305, USA; lisa.offringa@gmail.com; 2 Department of Public Health and Recreation, San José State University, San José, CA 95192, USA; jennifer.hartle@sjsu.edu; 3 Department of Biostatistics and Data Science, Wake Forest School of Medicine, Winston-Salem, NC 27157, USA; jrigdon@wakehealth.edu

**Keywords:** dietary fiber, healthy low-fat diet, healthy low-carbohydrate diet, weight loss, clinical trial

## Abstract

The daily intake of dietary fiber is well below the recommended levels in the US. The effect of adopting a low-fat vs. a low-carbohydrate weight loss diet on fiber intake is of interest but not well-documented, especially when both approaches promote high-quality food choices. The objective of this paper is to compare the quantity and sources of dietary fiber between a healthy low-fat (HLF) vs. healthy low-carbohydrate (HLC) diet group when consumed over 12 months in a weight loss diet study. In this secondary analysis of the Diet Intervention Examining The Factors Interacting with Treatment Success (DIETFITS) study, the amount and sources of dietary fiber were examined in generally healthy adults, 18–50 years of age, Body Mass Index (BMI) 28–40 kg/m^2^, randomized to HLF or HLC for 12 months, who had available 24-h recalls at 0 (*n* = 609), 3 (*n* = 549), 6 (*n* = 491), and 12 (*n* = 449) months. The dietary intake was estimated by the Nutrition Data System for Research (NDS-R). The sources of fiber were determined for the major food groups. Significantly more total dietary fiber was consumed by HLF at every post-randomization time point, and, at 12 m, was 23.04 ± 9.43 g vs. 18.61 ± 8.12 g for HLF vs. HLC, respectively, *p* < 0.0001. In both diet groups at 12 months, the highest amount of dietary fiber came from non-starchy vegetables (4.13 ± 3.05 g and 5.13 ± 3.59 g). The other primary sources of fiber at 12 months for the HLF group were from whole grains (3.90 ± 3.13 g) and fruits (3.40 ± 2.87 g), and, for the HLC group, were from plant protein and fat sources, such as nuts and seeds, their butters, and avocados (2.64 ± 2.64 g). In the DIETFITS study, the difference in the total fiber intake for the HLF vs. HLC groups was more modest than expected. The HLC group consumed reasonably high amounts of fiber from high-protein and high fat plant-based sources.

## 1. Introduction

Studies show that high-fiber intakes are associated with reduced rates of cardiovascular disease, cancer, inflammation, diabetes, and obesity [1,2,3,4]. Despite this, the fiber intake in the US is substantially below the recommendation of the 2020–2025 Dietary Guidelines for Americans. It is a nutrient of concern, with 90 percent of women and 97 percent of men eating less dietary fiber than recommended [5,6]. The recommended amount for adults 19–30 is 34 g/day for men, and 28 g/day for women [6]. The major food group sources of fiber in the US diet are grains, vegetables, legumes, fruits, nuts, and seeds [7]. According to the National Health and Nutrition Examination Survey (NHANES) from 2001 to 2010, the most common sources of dietary fiber in the US are grain products; however, only 10% of the grain intake reported was from whole grain sources [8,9].

The general approaches to low-fat vs. low-carb diets, two of the most commonly practiced approaches to weight loss, involve distinct differences regarding the food groups that are the main contributors to dietary fiber [10,11]. High-fiber foods such as legumes, grains, and fruits are low in fat, while high-fiber foods such as nuts and seeds are high in fat. Avocados, botanically a fruit, are high in fat, a good source of fiber, and are differentially recommended to be avoided or included for low-fat vs. low-carb diets, respectively. Whole grain foods are significantly higher in fiber than refined grains. While both of these are low in fat content, a greater public health benefit is placed on whole grains [12]. Vegetables are low in fat, and, although they are generally promoted in the low-carb diet approaches provided, this specifically refers to non-starchy vegetables; low-fat diet approaches tend not to differentiate starchy from non-starchy vegetables. While all sources of dietary fibers are plant foods, there are important differences in the fat content of plant foods, suggesting that, while shifting to a low-fat vs. a low-carb weight loss diet, an expected consequence would be differential changes in the sources and types of fiber, and different impacts on total fiber intake, that are not well characterized.

Our analysis included data from the Diet Intervention Examining the Factors Interacting with Treatment Success (DIETFITS) weight loss study, a 12-month intervention that utilized a healthy low-fat (HLF) vs. a healthy low-carb (HLC) approach to weight loss in generally healthy adults who were overweight or obese [13]. Both approaches promoted quality: choosing whole foods over processed foods, maximizing vegetable intake, and minimizing or avoiding refined grains [14]. The instructions for legumes, grains, fruits, nuts, and seeds differed by fat content (i.e., legumes and grains were encouraged for HLF, nuts and seeds were encouraged for HLC). The objective of this secondary analysis of the DIETFITS study was to examine and compare the changes in fiber intake from the baseline, and to examine the total amounts and sources of dietary fiber in the two diet groups. We hypothesized that the total fiber intake would be greater for the HLF group but that the differences in the types of fiber intake from the major food groups would vary by food groups for HLF vs. HLC as to which was higher.

## 2. Materials and Methods

### 2.1. Subjects

Participants in the DIETFITS study, a 12-month weight loss trial, were generally healthy adults, 18–50 years of age, with a BMI between 28–40 kg/m^2^ (Table 1). They were randomized to either HLF or HLC [14]. Figure S1 shows the main DIETFITS study participant flow chart. Changes in fiber intake used data from participants at baseline (*n* = 609), 3 months (*n* = 549), 6 months (*n* = 491), and 12 months (*n* = 449) in a linear mixed effects model. All study participants provided written informed consent. The study was approved by the Stanford University Human Subjects Committee [14]. This trial was registered at clinicaltrials.gov as NCT01826591 (15 September 2021).

### 2.2. Dietary Strategy

The diet strategy for DIETFITS has been described elsewhere in detail [14] and will be summarized here briefly. The overall approach was for participants to lower the fat or the carbohydrate content of their diets to the greatest extent possible with two general guidelines for both groups: (1) to maintain a focus on high-quality food choices, and (2) to achieve the lowest level of fat or carbohydrate restriction that could conceivably be maintained long-term, beyond the termination of the study. Notably, there was no specific calorie restriction guideline, and no specific target for an amount or percentage of fat or carbohydrates required by participants; the approach was to achieve ambitious and substantive changes that were also realistic.

The intervention involved twenty-two evening group classes led by study Health Educators. Both groups received similar instructions to choose whole foods over processed foods, to include vegetables, and to avoid refined grains and added sugars. For the first eight weeks of the study, HLF participants were instructed to strictly avoid all major dietary sources of fat. After the first eight weeks, when participants were working to design their own long-term, sustainable dietary strategies, they were allowed to slowly add limited amounts of fat to their diet while still maintaining the lowest fat content possible. HLF participants were instructed to consume whole grains, including steel cut oats, barley, quinoa, brown rice, and wild rice, and to choose a wide range of legumes, fresh fruit, and also low-fat dairy products. Similarly, for the first eight weeks of the study, HLC participants were instructed to avoid major sources of carbohydrates. Participants were instructed to consume nuts, seeds, avocados, and other sources of dietary fat, such as fatty fish, full fat dairy, and oils. HLC participants were instructed to avoid all legumes, fruit, grains, and starchy vegetables. After the first eight weeks, when participants were working to design their own long-term, sustainable dietary strategies, they were allowed to slowly add limited amounts of these carbohydrate sources. 

### 2.3. Dietary Assessment

Dietary intake was assessed using 3 unannounced 24-h multiple-pass dietary recall interviews (2 on weekdays and 1 on a weekend day) within a two-week window at four data collection time points (baseline, 3 M, 6 M, 12 M) for a total of 12 recalls. To determine shifts in sources of dietary fiber based on dietary assignment and changes in eating behavior, we investigated food categories modified from the University of Minnesota Nutrition Coordinating Center (NCC) Food Group Serving Count System consumed over the study period (Table S1). Data were collected using Nutrition Data System for Research (NDSR) [15,16,17], a computer-based software application developed by the Nutrition Coordinating Center (NCC), University of Minnesota, Minneapolis, MN, USA.

### 2.4. Statistical Analysis

Demographic data are summarized using means (standard deviations) for continuous variables and *n* (%) for categorical variables by randomized diet (HLF or HLC). NDSR provided estimates of total fiber consumed and servings consumed from 168 food groups. The 168 food groups were aggregated into 10 categories: fruits, vegetables, legumes, starches, whole grains, some whole grains, refined grains, protein foods, fats, and beverages, as summarized in Table S1.

A linear regression model was employed to estimate fiber per serving in each of the 168 food groups. Then, total grams of fiber per 168 food group for each participant at each available timepoint was estimated by multiplying servings consumed and the estimated fiber per serving (from the linear regression model). Total grams of fiber for each participant at each timepoint for the 10 categories was estimated by summing total grams of fiber for the food groups corresponding to each category.

Change in fiber per category over time (baseline, 3, 6, and 12 months) by diet (HLF or HLC) was estimated using a linear mixed effects model with main effects for diet, time, and diet by time interaction, and a random effect for participant [18]. Linear mixed models are ideal for estimating change over time as they allow for incompletely observed data (include all measurements from participants who have some subset of baseline 3, 6, 12) while accounting for correlation induced by repeated measures within participants. Statistical analyses were carried out in R version 3.6.1 [19].

## 3. Results

### 3.1. Demographics

The baseline characteristics of the two diet groups are presented in Table 1 [13]. The two groups were comparable at the baseline across all the characteristics. Overall, the study included 57% women and 43% men. The participants were generally well-educated, and approximately 60% were non-Hispanic white. The second largest race/ethnic group was Hispanic (~20%). The average BMI was approximately 33 kg/m^2^.

### 3.2. Macronutrients

The main DIETFITS study reported that, after 12 months, the participants in the HLC group were ingesting a mean of 1697.1 (±32.1) Kcals, 132.4 (±4.2) g of carbohydrates and 86.2 (±2.0) g of fat. The HLF group averaged 1716.1 (±34.5) Kcals, 212.9 (±5.0) g of carbohydrates and 57.3 (±1.7) g of fat [14]. Further dietary data are available in the primary paper [13].

### 3.3. Fiber Intake

The results show that the baseline fiber intake for the combined 609 participants included in the analysis was 21.82 ± 9.20. The estimated fiber intakes at the baseline (*n* = 609), 3 months (*n* = 549), 6 months (*n* = 491), and 12 months (*n* = 449) were entered into the linear mixed effects model to estimate the change over time.

#### 3.3.1. Fiber Intake by Diet Group—Total and by Major Food Groups

From the baseline to 12 months, the HLF group increased their total fiber by +0.33 (−0.81, 1.47) g/day (in the context of reporting a decrease in the mean calorie intake of −452.68 [−524.48, −380.88]), while the HLC group decreased their fiber intake by −3.29 (−4.42, −2.15) g/day (in the context of reporting a decrease in the mean calorie intake of −531.94 [−603.75, −460.13]). The changes in the fiber intake per 1000 kcal/day were +3.13 (2.48, 3.77) and +1.14 (0.5, 1.79) for the HLF and HLC groups, respectively. The differences in the total fiber intake between the two diet arms were statistically significant at all three post-randomization time points, as presented in Table 2 (*p* < 0.001).

#### 3.3.2. Fiber Intake Change in Similar Direction

**Figure 1:**
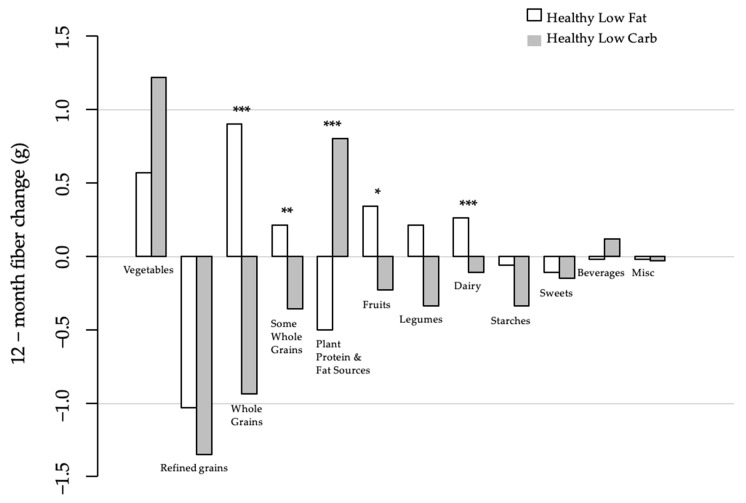
Differences in fiber intake in major food groups between HLF and HLC. * *p* < 0.05, ** *p* < 0.01, *** *p* < 0.0001.

Both groups increased the amount of fiber they ingested from non-starchy vegetables and decreased their intake of fiber from refined grains. Although not statistically significant, in absolute numbers, the increase at 12 months relative to the baseline for the HLC group was more than double that for the HLF group for fiber from non-starchy vegetables. At 12 months, the decrease in fiber from refined grains was similar and not significantly different between the HLF and HLC groups and was the largest source of decrease for both groups from any of the food categories, as seen in Figure 1. Fiber from starchy vegetables was lower in both groups at 12 months relative to the baseline and was not significantly different between the two diet groups.

#### 3.3.3. Fiber Intake Changes in Opposite Directions

At twelve months, the changes from the baseline in the categories of whole grains and some whole grains were higher for the HLF group and lower for the HLC group (Figure 1). For both the fruits and the legumes categories, there was higher fiber intake for the HLF group and lower for the HLC group. For the plant-based plant protein and fat sources category (includes nuts, seeds, avocado), the changes were in the opposite direction: lower for HLF and higher for HLC. The magnitude of the differential changes between the diet groups for all of these reached statistical significance with the exception of legumes.

#### 3.3.4. Miscellaneous Sources of Fiber Intake

Small amounts of fiber from a wide range of foods and beverages contributed modestly or negligibly to the overall fiber intake in both diet groups (See Table S2).

## 4. Discussion

In this secondary analysis of the DIETFITS 12-month weight loss study, the total fiber intake was consistently higher at 3, 6, and 12 months for the HLF diet group relative to the HLC diet group. The total fiber intake remained relatively stable for the HLF group, while it decreased for the HLC group during the study in the context of reduced calorie intake. In the context of energy-adjusted, rather than total, intake, the HLF group increased their fiber intake per 1000 kcal despite substantial reductions in the total and proportional carbohydrate intake, while the HLC group roughly maintained their fiber intake per 1000 kcal. Given that most of the major sources of dietary fiber are carbohydrate-rich foods, it was expected that the fiber intake would be higher in a higher vs. a lower carbohydrate diet. In this study, the total fiber difference between the two diet arms at 12 months, on average, was less than four grams, a relatively modest difference. However, closer investigation revealed that there were notable differences in the changes in the fiber intake for the specific food groups between the two diet groups; most prominently, the HLC group reported more than double the increase in fiber from vegetable intake compared to the HLF group, and increases in the fiber contribution from nuts, seeds, and avocados were significantly higher for HLC compared to HLF.

Given the increase in the vegetable fiber intake and the decrease in the refined grain fiber intake in both diet groups, this secondary analysis supports that participants were following the study instructions to follow both a healthy low-fat diet and healthy low-carb diet, respectively. As a consistent message throughout the study was to eat high-quality foods, most of the grains consumed were whole grains. Whole grains are a reliable source of dietary fiber, and many Americans consume grains as their primary source of fiber, but a diversity of dietary fiber from different plant sources is ideal for both gastrointestinal and overall health [20]. The sources of fiber for a low-fat diet would include whole wheat, which has numerous health benefits [21], but should also include diverse grains, such as oats [22], barley, and quinoa [23]. Foods containing refined grains have minimal amounts of dietary fiber and were generally discouraged during the study. Overall, both groups included vegetables as a major fiber source in their diet over the course of the study. Vegetables offer both dietary fiber and other important nutrients, and are recommended as a quality source of both, with strong associations with overall health benefits [24,25,26,27,28,29].

An emerging area of interest in the field of nutrition is the dietary regulation of the gut microbiome. A healthy microbiome is considered to play an important role in immune function and in preventing systemic inflammation [30,31]. One important factor in maintaining a healthy microbiome is to provide the gut bacteria with metabolically active carbohydrates. A high-fiber diet, complete with diverse microbiota accessible carbohydrates (MACs), can affect the condition of the intestinal microbiota [32,33].

Different types of dietary fiber have been linked to support for a healthy intestinal microbiota [34,35,36,37]. Specific dietary fibers may have differential effects on the microbiome. A better understanding of these diet–microbiome interactions may support dietary personalization to promote health [38,39,40]. Several studies demonstrate that different types of dietary fiber can have an impact on specific intestinal bacteria. As reported by our group previously, the initial shifts in the diets of the DIETFITS participants at 3 months showed diet-specific changes in their microbiome [41]. These changes might explain some of the substantial variability of the individual responses to low-fat vs. low-carbohydrate diets in general. This area merits further investigation.

This analysis has several strengths. First, the original DIETFITS study involves a relatively large sample size for a diet intervention, with high retention across multiple data collection time points over a 12-month period using an RCT design. Second, the study utilizes multiple interviewer-administered 24-h recalls using NDSR for diet data collection and database analysis purposes. While DIETFITS is not the first to analyze the differences between a low-carbohydrate and low-fat diet, this study is unique in its comparison of the fiber quantity and sources from the data obtained under these conditions.

There are also limitations to this analysis. Diet assessment that relies on self-reporting involves some degree of inaccuracy [42]. There is the possibility that the participants over-reported their intake of high-fiber foods as many of these foods are deemed “healthy” [43]. However, it is likely that this type of error would have been consistent within the individuals and between the diet groups, allowing for reasonably valid estimates of within- and between-group changes over time. An additional limitation involved the estimation of the servings of fiber per food category from the established settings in NDSR rather than determining these directly. The regression model used to estimate the servings of fiber in each of the specific food categories was the most feasible approach to this analysis given the data available.

## 5. Conclusions

Quality food sources of fiber can be found in both low-fat and low-carbohydrate diets. Throughout the 12-month DIETFITS study, the participants in both diet groups were encouraged to eat healthy, high-quality foods. The emphasis on quality during the study provides insight into the specific differential food sources of dietary fiber in low-fat vs. low-carbohydrate diets. The HLF diet group significantly increased their fiber intake throughout the study since they could include whole grains, which are a commonly consumed fiber source. These findings suggest that there are multiple strategies available for maximizing the intake of amounts and types of dietary fiber from the intake of whole and plant-based foods. A large proportion of fiber for both diet types was from non-starchy vegetables. In addition, the information from this analysis may be helpful in better understanding how to support a healthy microbiome through personalized nutrition, and how to help prevent and treat disease states through improved food choices.

## Figures and Tables

**Table 1:** Baseline demographics, and anthropometric and metabolic variables ^1^.

	HLF ^2^ (*n* = 305)	HLC ^3^ (*n* = 304)
**Sex**		
Women	167 (54.8%)	179 (58.9%)
Men	138 (45.2%)	125 (41.1%)
**Age**	39.3 (±6.8)	40.2 (±6.7)
**Education**		
Less than high school	2 (0.7%)	4 (1.3%)
High school graduate	5 (1.6%)	11 (3.6%)
Some college	63 (20.7%)	67 (22.0%)
College graduate or more	234 (76.7%)	221 (72.7%)
Missing	1 (0.3%)	1 (0.3%)
**Race/ethnicity** ^4^		
White	176 (57.7%)	182 (59.9%)
Hispanic	67 (22.0%)	61 (20.1%)
Asian	30 (9.8%)	30 (9.9%)
African American	10 (3.3%)	13 (4.3%)
AI/AN/PI	3 (1.0%)	0 (0.0%)
Other	19 (6.2%)	18 (5.9%)
**Weight (kg)**		
Women	90.7 (±11.5)	88.9 (±12.5)
Men	105.7 (±13.9)	106.8 (±13.7)
Total	97.5 (±14.7)	96.3 (±15.7)
**BMI (kg/m^2^)**		
Women	33.3 (±3.4)	32.9 (±3.4)
Men	33.5 (±3.4)	33.8 (±3.4)
Total	33.4 (±3.4)	33.3 (±3.4)
**Body fat (%)**		
Women	41.0 (±3.9)	40.4 (±4.0)
Men	29.9 (±4.5)	30.3 (±4.7)
Total	36.3 (±6.9)	36.5 (±6.6)
Missing	77 (25.2%)	66 (21.7%)

^1^ Values are mean ± SD for continuous variables, *n* (%) for categorical variables. ^2^ HLF = healthy low-fat diet. ^3^ HLC = healthy low-carbohydrate diet. ^4^ Race/ethnicity determined by self-report using fixed categories. AI = American Indian; AN = Alaskan Native; PI = Pacific Islander.

**Table 2:** Changes within and between group in fiber and fiber by food group. Estimate (95% CI) reported for all quantities of interest. Sample sizes for HLC and HLF at baseline: *n* = 304 and *n* = 305; at 3 months: *n* = 274 and *n* = 274; at 6 months: *n* = 251 and *n* = 240; and at 12 months: *n* = 224 and *n* = 225.

	HLC	HLF	HLC Minus HLF	*p*-Value ^1^
**Total fiber (g)**				
Baseline	21.63 (20.61, 22.65)	22.01 (21, 23.03)	−0.38 (−1.82, 1.06)	0.6049
3-month change	−5.7 (−6.76, −4.63)	2.07 (1.01, 3.13)	−7.77 (−9.27, −6.26)	<0.0001
6-month change	−4.62 (−5.72, −3.53)	1.46 (0.35, 2.58)	−6.09 (−7.65, −4.53)	<0.0001
12-month change	−3.29 (−4.42, −2.15)	0.33 (−0.81, 1.47)	−3.61 (−5.22, −2)	<0.0001
**Fiber per 1000** **calories (g/1000 kcal)**				
Baseline	10.1 (9.55, 10.65)	10.57 (10.01, 11.12)	−0.46 (−1.24, 0.32)	0.2451
3-month change	0.36 (−0.24, 0.97)	5.65 (5.05, 6.26)	−5.29 (−6.14, −4.43)	<0.0001
6-month change	0.9 (0.27, 1.52)	4.54 (3.91, 5.17)	−3.64 (−4.53, −2.76)	<0.0001
12-month change	1.14 (0.5, 1.79)	3.13 (2.48, 3.77)	−1.98 (−2.9, −1.07)	<0.0001
**Vegetables (g)**				
Baseline	3.79 (3.4, 4.18)	3.48 (3.09, 3.87)	0.31 (−0.24, 0.86)	0.274
3-month change	1.63 (1.17, 2.09)	1.01 (0.55, 1.47)	0.63 (−0.02, 1.28)	0.0586
6-month change	1.68 (1.2, 2.15)	1.29 (0.81, 1.77)	0.39 (−0.29, 1.06)	0.2598
12-month change	1.22 (0.73, 1.71)	0.57 (0.08, 1.06)	0.65 (−0.05, 1.34)	0.0673
**Refined grains (g)**				
Baseline	2.65 (2.46, 2.84)	2.91 (2.72, 3.1)	−0.26 (−0.53, 0)	0.0515
3-month change	−1.58 (−1.83, −1.34)	−0.79 (−1.04, −0.55)	−0.79 (−1.14, −0.44)	<0.0001
6-month change	−1.32 (−1.58, −1.07)	−0.68 (−0.94, −0.43)	−0.64 (−1, −0.28)	0.0004
12-month change	−1.35 (−1.61, −1.09)	−1.03 (−1.3, −0.77)	−0.31 (−0.68, 0.06)	0.0964
**Whole grains (g)**				
Baseline	2.13 (1.84, 2.41)	2.92 (2.64, 3.21)	−0.79 (−1.2, −0.39)	0.0001
3-month change	−1.21 (−1.58, −0.84)	0.57 (0.2, 0.94)	−1.78 (−2.3, −1.26)	<0.0001
6-month change	−0.81 (−1.19, −0.43)	0.65 (0.27, 1.04)	−1.46 (−2, −0.92)	<0.0001
12-month change	−0.94 (−1.33, −0.55)	0.9 (0.51, 1.29)	−1.84 (−2.39, −1.29)	<0.0001
**Some whole grains (g)**				
Baseline	0.76 (0.54, 0.97)	0.88 (0.66, 1.09)	−0.12 (−0.42, 0.19)	0.4516
3-month change	−0.55 (−0.8, −0.3)	0.15 (−0.1, 0.4)	−0.7 (−1.05, −0.35)	0.0001
6-month change	−0.5 (−0.76, −0.25)	0.19 (−0.07, 0.45)	−0.69 (−1.05, −0.32)	0.0002
12-month change	−0.36 (−0.63, −0.1)	0.21 (−0.06, 0.47)	−0.57 (−0.94, −0.19)	0.0029
**Protein/fat (g)**				
Baseline	1.8 (1.56, 2.04)	1.44 (1.2, 1.67)	0.36 (0.03, 0.7)	0.0346
3-month change	0.91 (0.61, 1.22)	−0.56 (−0.87, −0.26)	1.48 (1.05, 1.9)	<0.0001
6-month change	0.83 (0.52, 1.14)	−0.38 (−0.69, −0.06)	1.21 (0.76, 1.65)	<0.0001
12-month change	0.8 (0.48, 1.12)	−0.5 (−0.82, −0.17)	1.3 (0.84, 1.75)	<0.0001
**Fruits (g)**				
Baseline	2.64 (2.34, 2.94)	2.97 (2.67, 3.28)	−0.33 (−0.76, 0.1)	0.1288
3-month change	−0.37 (−0.73, −0.01)	0.71 (0.35, 1.08)	−1.08 (−1.6, −0.57)	<0.0001
6-month change	−0.35 (−0.72, 0.02)	0.38 (0, 0.76)	−0.73 (−1.26, −0.19)	0.0075
12-month change	−0.23 (−0.62, 0.16)	0.34 (−0.05, 0.72)	−0.57 (−1.11, −0.02)	0.0428
**Legumes (g)**				
Baseline	1.18 (0.87, 1.48)	2.14 (1.83, 2.44)	−0.96 (−1.4, −0.52)	<0.0001
3-month change	−0.47 (−0.86, −0.08)	−0.05 (−0.43, 0.34)	−0.42 (−0.97, 0.12)	0.1288
6-month change	−0.36 (−0.76, 0.04)	0.03 (−0.37, 0.43)	−0.39 (−0.95, 0.18)	0.1771
12-month change	−0.34 (−0.75, 0.07)	0.21 (−0.2, 0.62)	−0.55 (−1.13, 0.03)	0.0626
**Dairy (g)**				
Baseline	0.53 (0.43, 0.64)	0.66 (0.55, 0.76)	−0.13 (−0.27, 0.02)	0.0989
3-month change	−0.1 (−0.23, 0.03)	0.2 (0.06, 0.33)	−0.3 (−0.49, −0.11)	0.0021
6-month change	−0.15 (−0.29, −0.01)	0.34 (0.2, 0.48)	−0.49 (−0.68, −0.29)	<0.0001
12-month change	−0.11 (−0.25, 0.03)	0.26 (0.12, 0.4)	−0.37 (−0.57, −0.17)	0.0003
**Potatoes/starch (g)**				
Baseline	1.12 (0.98, 1.26)	1.09 (0.95, 1.24)	0.03 (−0.17, 0.23)	0.7849
3-month change	−0.44 (−0.63, −0.24)	−0.07 (−0.27, 0.12)	−0.36 (−0.63, −0.09)	0.0091
6-month change	−0.39 (−0.59, −0.2)	−0.25 (−0.45, −0.05)	−0.14 (−0.42, 0.14)	0.3148
12-month change	−0.34 (−0.55, −0.14)	−0.06 (−0.26, 0.14)	−0.28 (−0.57, 0)	0.0539
**Sweets (g)**				
Baseline	0.43 (0.37, 0.49)	0.39 (0.33, 0.45)	0.03 (−0.05, 0.12)	0.4531
3-month change	−0.16 (−0.24, −0.08)	−0.19 (−0.27, −0.1)	0.03 (−0.09, 0.15)	0.6253
6-month change	−0.14 (−0.22, −0.05)	−0.09 (−0.18, −0.01)	−0.05 (−0.17, 0.07)	0.4614
12-month change	−0.15 (−0.24, −0.06)	−0.11 (−0.2, −0.02)	−0.04 (−0.17, 0.08)	0.4983
**Beverages (g)**				
Baseline	1.06 (0.94, 1.18)	0.87 (0.75, 0.99)	0.19 (0.02, 0.36)	0.0311
3-month change	0.11 (0.01, 0.21)	−0.01 (−0.11, 0.09)	0.12 (−0.02, 0.26)	0.0876
6-month change	0.13 (0.02, 0.23)	0.04 (−0.06, 0.14)	0.08 (−0.06, 0.23)	0.2551
12-month change	0.12 (0.02, 0.23)	−0.02 (−0.13, 0.08)	0.15 (0, 0.3)	0.0531
**Miscellaneous (g)**				
Baseline	0.19 (0.11, 0.28)	0.2 (0.12, 0.29)	−0.01 (−0.13, 0.11)	0.8782
3-month change	−0.07 (−0.18, 0.05)	−0.04 (−0.15, 0.08)	−0.03 (−0.2, 0.13)	0.7128
6-month change	0.05 (−0.07, 0.17)	0.01 (−0.11, 0.14)	0.04 (−0.13, 0.21)	0.6753
12-month change	−0.03 (−0.16, 0.09)	−0.02 (−0.14, 0.11)	−0.02 (−0.19, 0.16)	0.8619

^1^ Confidence intervals and *p*-values from linear mixed effects model for outcome as a function of time, diet, and time × diet interaction. No multiple comparison adjustment performed.

## Data Availability

Data described in the manuscript, code book, and analytic code will be made available upon request pending application and approval by the corresponding author.

## Supplementary Materials

The following are available online at https://www.mdpi.com/article/10.3390/nu13103625/s1, Figure S1: Main DIETFITS Study Participant Flowchart, Table S1: Food Groups modified from University of Minnesota Nutrition Coordinating Center Food Group Serving Count System, Table S2: Estimated Fiber Intake (g) at 12 months by Diet and Food Group.
