# Supplementary material for: Changes in Quantity and Sources of Dietary Fiber from Adopting Healthy Low-Fat vs. Healthy Low-Carb Weight Loss Diets: Secondary Analysis of DIETFITS Weight Loss Diet Study
Source: Nutrients. 2021 Oct 16;13(10):3625. doi: 10.3390/nu13103625 (PMC8539701; doi:10.3390/nu13103625)

Supporting Material

**Table S1: Food Groups modified from University of Minnesota Nutrition Coordinating Center Food Group Serving Count System**

| Food group | Component foods |
|---|---|
| vegetables | darkgreen_veg<br>deepyellow_veg<br>other_veg<br>veg_juice<br>veg_savory_snack<br>fried_veg |
| refined_grains | refined_grain_mix<br>refined_grain_bread<br>refined_grain_other<br>refined_grain_cracker<br>refined_grain_pasta<br>refined_grain_cereal_nosweet<br>refined_grain_cereal_sweet<br>refined_grain_cookie<br>refined_grain_bar<br>refined_grain_chip |
| whole_grains | whole_grain_mix<br>whole_grain_bread<br>whole_grain_other<br>whole_grain_cracker<br>whole_grain_pasta<br>whole_grain_cereal_nosweet<br>whole_grain_cereal_sweet<br>whole_grain_cookie<br>whole_grain_chip<br>whole_grain_bar<br>popcorn popcorn_flavored |
| some_whole_grains | some_whole_grain_mix<br>some_whole_grain_bread<br>some_whole_grain_other<br>some_whole_grain_cracker<br>some_whole_grain_pasta<br>some_whole_grain_cereal_nosweet<br>some_whole_grain_cereal_sweet<br>some_whole_grain_cookie<br>some_whole_grain_chip<br>some_whole_grain_bar |
| protein_fat | nuts_seeds<br>nut_seed_butters<br>meat_substitute |

Supporting Material

| | |
|---|---|
| | avocado |
| fruits | citrus_juice<br>fruit_juice_nocitrus<br>citrus_fruit<br>fruit_nocitrus<br>tomato<br>fried_fruit<br>fruit_savory_snack |
| Legumes | legumes |
| dairy | milk_whole<br>milk_reduced_fat<br>milk_low_fat<br>milk_nondairy<br>milk_flavored_whole<br>milk_flavored_reduced_fat<br>milk_flavored_low_fat<br>sweetened_nonfat_dry_milk<br>artificially_sweetened_nonfat_dry_milk<br>sweetened_no_nonfat_dry_milk<br>artificially_sweetened_no_nonfat_dry_milk<br>cheese_full_fat cheese_reduced_fat<br>cheese_low_fat cheese_nondairy<br>yogurt_sweetened_whole<br>yogurt_sweetened_low<br>yogurt_sweetened_free<br>yogurt_artificially_sweetened_whole<br>yogurt_artificially_sweetened_low<br>yogurt_artificially_sweetened_free<br>yogurt_nondairy frozen_dairy<br>frozen_nondairy pudding_other<br>artificially_sweetened_pudding<br>dairy_sweetened_replacement<br>dairy_artificially_sweetened_replacement |
| potatoes_starch | white_potatoes<br>fried_potatoes<br>starchy_veg |
| sweets | sugar<br>syrup_honey<br>sauces_sweet<br>sauces_sweet_regular<br>sauces_regular<br>sauces_reduced<br>chocolate_candy |

| | |
|---|---|
| | nonchocolate_candy<br>frosting<br>sugar_substitute |
| beverages | sweetened_coffee<br>artificially_sweetened_coffee<br>unsweetened_coffee |
| misc | gravy<br>gravy_reduced<br>nongrain_flour<br>soup_broth<br>pickled_foods<br>misc_dessert<br>sauces_regular<br>sauces_reduced<br>nondairy_sweetened_meal_replacement<br>nondairy_artificially_sweetened_meal_replacement<br>nondairy_unsweetened_meal_replacement |

Supporting Material

**Table S2: Estimated fiber intake (g) at 12 months by diet and food group**

| | Healthy low carb n=224 | Healthy low fat n=225 |
|---|---|---|
| **Vegetables** | | |
| darkgreen_veg | 1.92 (±1.95) | 1.20 (±1.33) |
| deepyellow_veg | 0.85 (±1.62) | 0.88 (±1.51) |
| other_veg | 2.27 (±2.00) | 1.83 (±1.63) |
| veg_juic | 0.03 (±0.25) | 0.07 (±0.49) |
| veg_savory_snack | 0.06 (±0.23) | 0.15 (±0.84) |
| fried_veg | 0.01 (±0.04) | 0.00 (±0.04) |
| **Refined grains** | | |
| refined_grain_mix | 0.27 (±0.41) | 0.46 (±0.53) |
| refined_grain_brea | 0.34 (±0.52) | 0.52 (±0.63) |
| refined_grain_other | 0.22 (±0.50) | 0.36 (±0.62) |
| refined_grain_cracker | 0.03 (±0.17) | 0.05 (±0.22) |
| refined_grain_past | 0.16 (±0.84) | 0.22 (±0.47) |
| refined_grain_cereal_nosweet | 0.00 (±0.00) | 0.00 (±0.01) |
| refined_grain_cereal_sweet | 0.00 (±0.00) | 0.00 (±0.02) |
| refined_grain_cooki | 0.13 (±0.30) | 0.18 (±0.36) |
| refined_grain_bar | 0.09 (±0.45) | 0.05 (±0.29) |
| refined_grain_chip | 0.02 (±0.17) | 0.05 (±0.21) |
| **Whole grains** | | |
| whole_grain_mix | 0.32 (±0.99) | 1.59 (±2.29) |
| whole_grain_brea | 0.18 (±0.70) | 0.62 (±1.23) |
| whole_grain_other | 0.32 (±0.83) | 0.56 (±1.10) |
| whole_grain_cracker | 0.04 (±0.25) | 0.05 (±0.38) |
| whole_grain_past | 0.06 (±0.42) | 0.28 (±1.03) |
| whole_grain_cereal_nosweet | 0.07 (±0.35) | 0.25 (±0.71) |
| whole_grain_cereal_sweet | 0.04 (±0.27) | 0.24 (±0.73) |
| whole_grain_cooki | 0.00 (±0.07) | 0.02 (±0.18) |
| whole_grain_chip | 0.07 (±0.30) | 0.16 (±0.43) |
| whole_grain_bar | 0.01 (±0.06) | 0.04 (±0.20) |
| popcorn | 0.05 (±0.23) | 0.10 (±0.39) |
| popcorn_flavore | 0.00 (±0.00) | 0.00 (±0.01) |
| **Some whole grains** | | |
| some_whole_grain_mix | 0.02 (±0.30) | 0.07 (±0.41) |
| some_whole_grain_brea | 0.08 (±0.29) | 0.35 (±0.99) |
| some_whole_grain_other | 0.02 (±0.30) | 0.03 (±0.24) |
| some_whole_grain_cracker | 0.00 (±0.03) | 0.00 (±0.03) |
| some_whole_grain_past | 0.00 (±0.00) | 0.00 (±0.00) |
| some_whole_grain_cereal_nosweet | 0.07 (±0.85) | 0.29 (±2.06) |
| some_whole_grain_cereal_sweet | 0.02 (±0.26) | 0.09 (±0.51) |

Supporting Material

| | Healthy low carb | Healthy low fat |
|---|---|---|
| some_whole_grain_cooki | 0.02 (±0.10) | 0.02 (±0.09) |
| some_whole_grain_chip | 0.00 (±0.03) | 0.02 (±0.13) |
| some_whole_grain_bar | 0.17 (±0.95) | 0.25 (±1.20) |
| **Protein/fat** | | |
| nuts_seeds | 1.37 (±2.09) | 0.47 (±0.87) |
| nut_seed_butters | 0.18 (±0.53) | 0.09 (±0.23) |
| meat_substitut | 0.06 (±0.27) | 0.06 (±0.20) |
| avocado | 1.03 (±1.53) | 0.32 (±0.89) |
| **Fruits** | | |
| citrus_juic | 0.13 (±0.43) | 0.14 (±0.36) |
| fruit_juice_nocitrus | 0.00 (±0.00) | 0.00 (±0.00) |
| citrus_fruit | 0.38 (±1.10) | 0.79 (±1.88) |
| fruit_nocitrus | 0.93 (±1.51) | 1.55 (±1.64) |
| tomato | 0.97 (±1.00) | 0.91 (±0.97) |
| fried_fruit | 0.00 (±0.05) | 0.00 (±0.00) |
| fruit_savory_snack | 0.01 (±0.11) | 0.01 (±0.10) |
| legumes | 0.85 (±1.87) | 2.39 (±3.15) |
| **Dairy** | | |
| milk_whol | 0.00 (±0.00) | 0.00 (±0.00) |
| milk_reduced_fat | 0.00 (±0.00) | 0.00 (±0.00) |
| milk_low_fat | 0.03 (±0.11) | 0.22 (±0.34) |
| milk_nondairy | 0.17 (±0.47) | 0.14 (±0.55) |
| milk_flavored_whol | 0.00 (±0.00) | 0.00 (±0.00) |
| milk_flavored_reduced_fat | 0.00 (±0.00) | 0.01 (±0.16) |
| milk_flavored_low_fat | 0.00 (±0.02) | 0.00 (±0.00) |
| sweetened_nonfat_dry_milk | 0.07 (±0.52) | 0.07 (±0.43) |
| artificially_sweetened_nonfat_dry_milk | 0.00 (±0.00) | 0.00 (±0.06) |
| sweetened_no_nonfat_dry_milk | 0.00 (±0.05) | 0.01 (±0.08) |
| artificially_sweetened_no_nonfat_dry_milk | 0.00 (±0.00) | 0.00 (±0.00) |
| cheese_full_fat | 0.00 (±0.00) | 0.00 (±0.00) |
| cheese_reduced_fat | 0.01 (±0.03) | 0.02 (±0.03) |
| cheese_low_fat | 0.00 (±0.00) | 0.00 (±0.00) |
| cheese_nondairy | 0.00 (±0.00) | 0.00 (±0.00) |
| yogurt_sweetened_whol | 0.02 (±0.10) | 0.00 (±0.06) |
| yogurt_sweetened_low | 0.02 (±0.11) | 0.02 (±0.11) |
| yogurt_sweetened_fre | 0.02 (±0.14) | 0.24 (±0.69) |
| yogurt_artificially_sweetened_whol | 0.00 (±0.00) | 0.00 (±0.00) |
| yogurt_artificially_sweetened_low | 0.01 (±0.07) | 0.03 (±0.15) |
| yogurt_artificially_sweetened_fre | 0.05 (±0.17) | 0.16 (±0.38) |
| yogurt_nondairy | 0.00 (±0.00) | 0.00 (±0.00) |
| frozen_dairy | 0.00 (±0.00) | 0.00 (±0.00) |
| frozen_nondairy | 0.00 (±0.01) | 0.00 (±0.01) |

Supporting Material

| | Healthy low carb | Healthy low fat |
|---|---|---|
| pudding_other | 0.00 (±0.00) | 0.00 (±0.00) |
| artificially_sweetened_pudding | 0.01 (±0.08) | 0.01 (±0.08) |
| dairy_sweetened_replacement | 0.00 (±0.00) | 0.00 (±0.00) |
| dairy_artificially_sweetened_replacement | 0.01 (±0.13) | 0.01 (±0.19) |
| **Potatoes/starches** | | |
| white_potatoes | 0.21 (±0.47) | 0.30 (±0.57) |
| fried_potatoes | 0.21 (±0.53) | 0.33 (±0.86) |
| starchy_veg | 0.35 (±0.81) | 0.41 (±0.88) |
| **Sweets** | | |
| sugar | 0.02 (±0.04) | 0.03 (±0.06) |
| syrup_honey | 0.00 (±0.00) | 0.00 (±0.00) |
| sauces_sweet | 0.00 (±0.00) | 0.00 (±0.00) |
| sauces_sweet_regular | 0.03 (±0.15) | 0.04 (±0.20) |
| chocolate_candy | 0.15 (±0.39) | 0.13 (±0.35) |
| nonchocolate_candy | 0.00 (±0.00) | 0.00 (±0.00) |
| frosting | 0.00 (±0.00) | 0.00 (±0.00) |
| sugar_substitut | 0.05 (±0.16) | 0.04 (±0.15) |
| **Beverages** | | |
| sweetened_coffe | 0.00 (±0.00) | 0.00 (±0.00) |
| artificially_sweetened_coffe | 0.00 (±0.00) | 0.00 (±0.00) |
| unsweetened_coffe | 1.17 (±1.24) | 0.85 (±0.98) |
| **Miscellaneous** | | |
| gravy | 0.00 (±0.00) | 0.00 (±0.00) |
| gravy_reduce | 0.01 (±0.11) | 0.00 (±0.03) |
| nongrain_flour | 0.03 (±0.36) | 0.01 (±0.08) |
| soup_broth | 0.02 (±0.06) | 0.03 (±0.07) |
| pickled_foods | 0.00 (±0.00) | 0.00 (±0.00) |
| misc_dessert | 0.00 (±0.00) | 0.00 (±0.00) |
| sauces_regular.1 | 0.02 (±0.10) | 0.04 (±0.16) |
| sauces_reduce.1 | 0.00 (±0.00) | 0.00 (±0.00) |
| nondairy_sweetened_meal_replacement | 0.00 (±0.00) | 0.00 (±0.01) |
| nondairy_artificially_sweetened_meal_replacement | 0.00 (±0.01) | 0.00 (±0.01) |
| nondairy_unsweetened_meal_replacement | 0.07 (±0.69) | 0.09 (±0.50) |

Supporting Material

**Figure S1: Main DIETFITS Study Participant Flowchart**

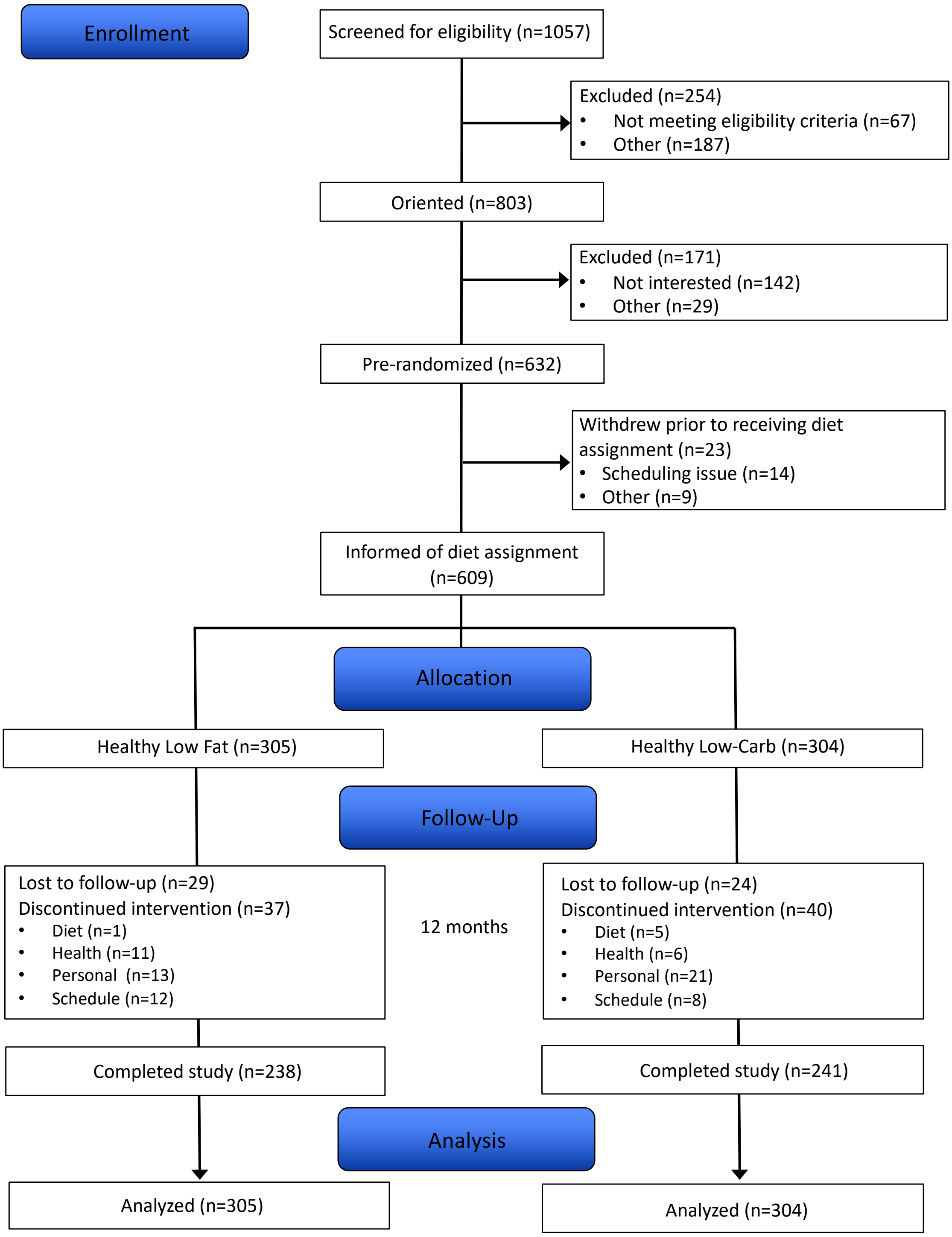

Supplement: Supplementary file 1 [file nutrients-13-03625-s001.zip › nutrients-1420128-supplementary.pdf]
